# Critical role of parasite‐mediated energy pathway on community response to nutrient enrichment

**DOI:** 10.1002/ece3.9622

**Published:** 2022-12-13

**Authors:** Patch Thongthaisong, Minoru Kasada, Hans‐Peter Grossart, Sabine Wollrab

**Affiliations:** ^1^ Department of Plankton and Microbial Ecology Leibniz Institute of Freshwater Ecology and Inland Fisheries (IGB) Berlin Germany; ^2^ Institute for Biochemistry and Biology Potsdam University Potsdam Germany; ^3^ Graduate School of Life Sciences Tohoku University Sendai Japan; ^4^ Berlin‐Brandenburg Institute of Advanced Biodiversity Research (BBIB) Berlin Germany

**Keywords:** adaptive preference, energy flow, food web, mycoloop, parasite‐mediated energy pathway, parasites, parasitic fungi

## Abstract

Parasites form an integral part of food webs, however, they are often ignored in classic food web theory or limited to the investigation of trophic transmission pathways. Specifically, direct consumption of parasites by nonhost predators is rarely considered, while it can contribute substantially to energy flow in food webs. In aquatic systems, chytrids constitute a major group of fungal parasites whose free‐living infective stages (zoospores) form a highly nutritional food source to zooplankton. Thereby, the consumption of zoospores can create an energy pathway from otherwise inedible phytoplankton to zooplankton (“mycoloop”). This parasite‐mediated energy pathway might be of special importance during phytoplankton blooms dominated by inedible or toxic primary producers like cyanobacteria, which are on the rise with eutrophication and global warming. We theoretically investigated community dynamics and energy transfer in a food web consisting of an edible nonhost and an inedible host phytoplankton species, a parasitic fungus, and a zooplankton species grazing on edible phytoplankton and fungi. Food web dynamics were investigated along a nutrient gradient contrasting nonadaptive zooplankton species representative for filter feeders like cladocerans and zooplankton with the ability to actively adapt their feeding preferences like many copepod species. Overall, the importance of the mycoloop for zooplankton increases with nutrient availability. This increase is smooth for nonadaptive consumers. For adaptive consumers, we observe an abrupt shift from an almost exclusive preference for edible phytoplankton at low nutrient levels to a strong preference for parasitic fungi at high nutrient levels. The model predicts that parasitic fungi could contribute up to 50% of the zooplankton diet in nutrient‐rich environments, which agrees with empirical observations on zooplankton gut content from eutrophic systems during blooms of inedible diatoms or cyanobacteria. Our findings highlight the role of parasite‐mediated energy pathways for predictions of energy flow and community composition under current and future environmental change.

## Graphical abstract

Parasites form an integral part of food webs, however, mechanistic insights into the role of parasites for energy flow and community dynamics are currently limited by a lack of conceptual studies investigating host–parasite interactions in a community context, including nonhost species. We theoretically investigated community dynamics and energy transfer in a food web, where direct consumption of the free‐living state of a parasite creates an energy pathway to a predator from an otherwise inedible host. The model predicts that parasitic fungi can contribute up to 50% of the zooplankton diet in nutrient‐rich environments, agreeing with empirical observations on zooplankton gut content from eutrophic systems during cyanobacterial or large diatom blooms.

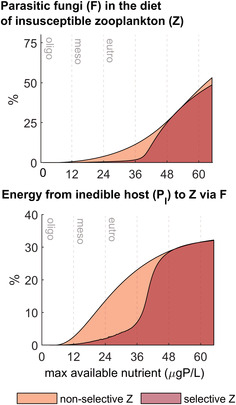

## 1 INTRODUCTION

Parasites are an integral but often neglected part of food webs (Lafferty et al., 2008; Sukhdeo, 2012). Adding parasites to classic trophic (predator–prey) interaction networks increases biodiversity and food web complexity (Morton et al., 2021). Thereby, parasite–host interactions are closely interwoven with classic trophic interactions, not only as a part of trophic transmission pathways but also consumption of parasites by nonhost consumers. In both cases, parasites can be consumed via concomitant link (consumption of an infected host) or directly (Johnson et al., 2010). Interestingly, direct consumption of ectoparasites or parasite propagules (the free‐living infectious stages) can create an energy pathway from otherwise inedible hosts to a nonhost consumer (Banerji et al., 2015; Miki et al., 2011; Prosnier et al., 2018). Examples of such parasite‐mediated energy pathways (see review by Johnson et al., 2010) are ticks on mammal skin that are eaten by birds (Ndlovu & Combrink, 2015), earthworms feeding on parasitic flatworms on snail skin (Hobart et al., 2021), cleaner fish feeding on ectoparasites of coral reef fish (Waldie et al., 2011), and zooplankton consumption of zoospores (the propagules of parasitic chytrids) emerging from infected phytoplankton cells (Kagami et al., 2014) or from the skin of amphibians (Buck et al., 2011).

Empirical studies suggest that parasite consumption by nonhost predators can substantially contribute to energy flow in food webs (Grutter et al., 2003; Michalska‐Smith et al., 2018; Rasconi et al., 2014) and influence community composition (Grutter et al., 2003; Waldie et al., 2011). However, mechanistic insight on the energetic role of parasites, their influence on nonhost species, and resulting consequences for community responses to environmental change are currently limited by a lack of conceptual food web studies that go beyond trophic transmission pathways (but see Miki et al., 2011, Buck et al., 2015, Prosnier et al., 2018). In this study, we investigate the role of direct consumption of parasites by nonhost consumers. We focus on an example from the aquatic environment where zooplankton consumption of zoospores of parasitic fungal (chytrids) can create an additional energy pathway from otherwise inedible host phytoplankton to zooplankton, the so‐called “mycoloop” (Kagami et al., 2007; Miki et al., 2011). Parasitic chytrids form a dominant group of parasites in aquatic systems (Grossart et al., 2019; Lefèvre et al., 2012). Several studies have shown that zoospores can form a highly nutritional food source for zooplankton, providing high concentrations of polyunsaturated fatty acids (PUFAs; Agha et al., 2016; Kagami et al., 2007; Rasconi et al., 2020). Although edible phytoplankton is the primary food source of zooplankton, zoospores might form an important alternative food source during phytoplankton blooms dominated by inedible species (Gsell et al., 2022; Rasconi et al., 2014). In temperate regions, the mycoloop might regularly form an important energy source for zooplankton during the late summer season, when phytoplankton communities are typically dominated by less edible algae (Sommer et al., 2012; Van den Wyngaert et al., 2022), but also during spring blooms if dominated by cyanobacteria (Rasconi et al., 2012). Furthermore, its importance may be on the rise with worldwide eutrophication and global warming leading to the increasing dominance of often inedible or even toxic cyanobacteria (Bogard et al., 2020; Huisman et al., 2018). Energy flow along the mycoloop might also have indirect effects on other edible/nonhost primary producers via apparent or resource competition (Kagami et al., 2014; Miki et al., 2011).

**FIGURE 1:**
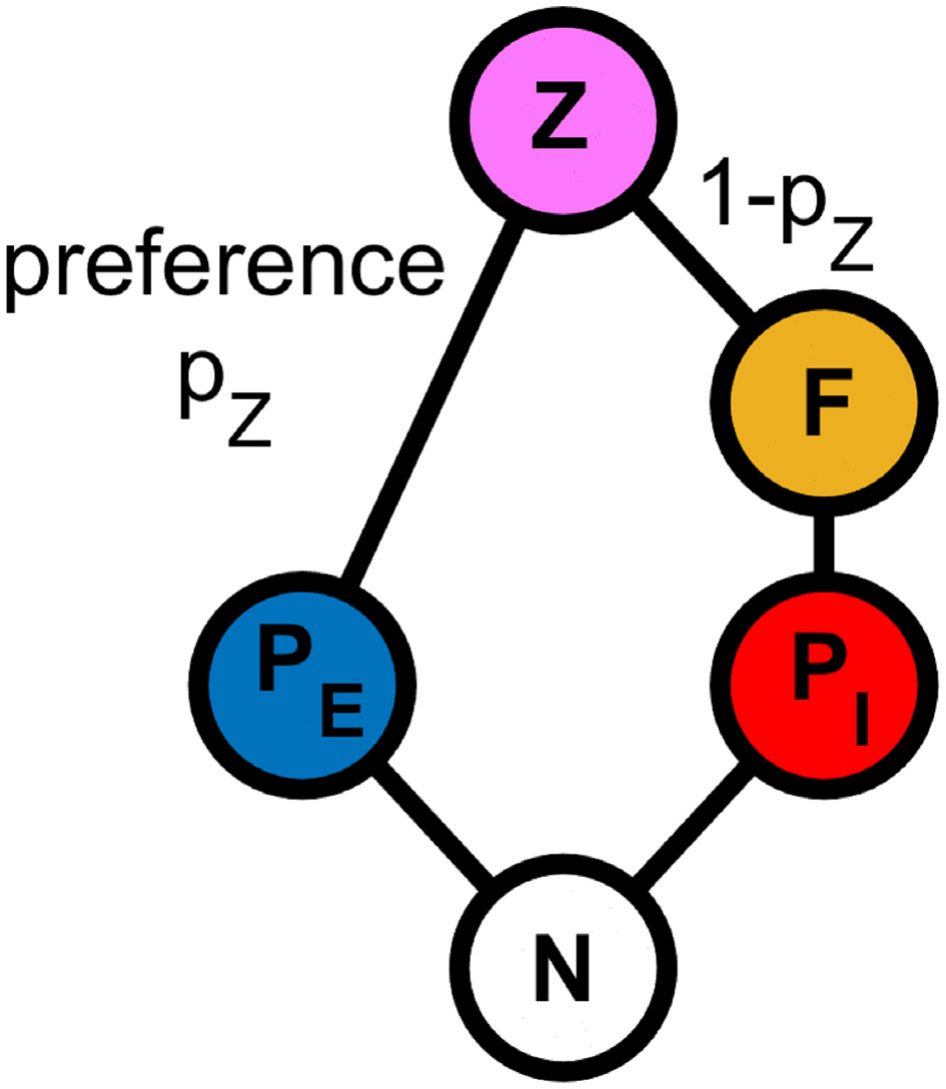
Planktonic model food web based on the following assumptions: One phytoplankton species *P*_*E*_ is well‐edible for zooplankton *Z*, whereas the other phytoplankton species *P*_*I*_ is inedible for *Z*. Both phytoplankton species compete for a shared resource *N*, with *P*_*E*_ (*P*_*I*_) being the superior (inferior) resource competitor. Furthermore, *P*_*E*_ is insusceptible to fungal infection, whereas *P*_*I*_ is susceptible to infection by *F*. *F* is edible for *Z*, which is insusceptible to fungal infection, thereby creating an alternative nutrient pathway from the otherwise inedible *P*_*I*_ to *Z*, that is, the mycoloop. The feeding preference of *Z* for *P*_*E*_ versus *F* is defined by *p*_*Z*_ and (1−*p*_*Z*_), respectively.

The direct and indirect effects of the mycoloop on the plankton community have been theoretically investigated by Miki et al. (2011) using a food web consisting of two groups of phytoplankton, edible nonhost versus inedible host, which compete for a shared resource, parasitic fungi specialized on inedible host phytoplankton, and zooplankton feeding on edible nonhost phytoplankton and parasitic fungi. In this mycoloop food web, there are two alternative energy pathways to zooplankton: one from edible nonhost phytoplankton and the other from parasitic fungi emerging from inedible host phytoplankton (mycoloop; see Figure 1). The theoretical study by Miki et al. (2011) has shown that chytrid infection of inedible phytoplankton species can indirectly support edible nonhost phytoplankton species via decreasing resource competition, however, zooplankton consumption of zoospores may at the same time lead to increasing top‐down pressure on edible phytoplankton (Kagami et al., 2014; Miki et al., 2011). Due to the multiple feedbacks within the plankton community, the net effects of parasitic fungi on primary production, community composition, and energy transfer are difficult to predict. This gets even more complex if nonlinear food uptake rates are considered (Wollrab et al., 2015).

In this study, we, therefore, extend the investigations of Miki et al. (2011) by taking nonlinear, saturating food uptake rates and the possibility of different zooplankton prey preferences into account. Furthermore, we distinguish two major feeding guilds of the zooplankton community, that is, passive filter feeders like cladocerans (Uszko et al., 2015) versus active hunters like copepods (Meunier et al., 2016). Especially for copepods, experimental results indicate that they might actively choose fungi over other (less nutritious) prey (Ray et al., 2016).

Our results show that under the assumption of saturating food/nutrient uptake rates, the increasing importance of the mycoloop with nutrient enrichment is much more pronounced than under the assumption of linear food uptake rates, with predictions being closer to empirical observations (Rasconi et al., 2014). Furthermore, the importance of the mycoloop increases smoothly for a nonadaptive consumer, while we observe an abrupt shift toward a strong preference for parasitic fungi from low to high nutrient levels for zooplankton with adaptive prey preference. Our theoretical results emphasize the importance of the parasite‐mediated energy pathway on community dynamics and trophic transfer efficiency and how this is modulated by consumer feeding strategies.

## 2 METHODS

### 2.1 Model system

We investigated the biomass distribution and dynamics of a planktonic model food web consisting of two types of primary producers (phytoplankton) that share a limiting nutrient source *N*, assumed to be phosphorus (μgP L^−1^), a zooplankton species *Z*, and parasitic fungi *F*. The food web is based on the following assumptions: one phytoplankton species *P*_*E*_ is well‐edible for *Z*, whereas the other phytoplankton species *P*_*I*_ is inedible for *Z*. *P*_*E*_ is the superior resource competitor, whereas *P*_*I*_ is the inferior resource competitor. Furthermore, *P*_*E*_ is insusceptible to fungal infection, whereas *P*_*I*_ is susceptible to infection by *F*. *F* is edible for *Z*, which is insusceptible to fungal infection, thereby creating an alternative nutrient pathway from the otherwise inedible *P*_*I*_ to *Z*, that is, the mycoloop (Figure 1).

The food web was translated into a corresponding differential equation system (Equations (1), (2), (3), (4), (5)). All biomasses are expressed in units of phosphorus (μgP L^−1^). Nutrient dynamics were assumed to follow chemostat dynamics with maximum nutrient availability *N*_max_ and dilution rate *q* (Equation 1). In contrast to the basic model presented in Miki et al. (2011), we assumed a saturating functional response for the nutrient uptake of both phytoplankton species following Monod kinetics with maximum growth rate $\mu_{\max,i},i\in \left\{E,I\right\}$, and half saturation constant *K* (Equations 2 and 3). Similar to Miki et al. (2011) and in line with published dependencies of infection rate on host density (Frenken et al., 2020; Gerla et al., 2013), we assumed a linear dependency of the infection of *F* on host biomass *P*_*I*_ with infection rate *β* and conversion efficiency *f*_*F*_ (Equation 4). We furthermore assumed a saturating functional response type III (Holling, 1959) for the food uptake term of *Z* with food uptake rate *a*_*Z*_ and handling time $h_{i},i\in \left\{P_{E},F\right\}$ (Equation 5). A functional response type III has been shown to be representative of zooplankton species with selective feeding behavior, like raptorial copepods, but also for filter feeders with the ability to downregulate their filtration rate if prey density is low, which has been reported for several daphnia species (Kiørboe et al., 2018; Sandhu et al., 2019; Uszko et al., 2015). This functional response type specifically allows to mimic the (disproportional) release of low abundant prey from predation pressure relevant for both raptorial predators as well as filter feeders, which is not captured by a functional response type II (Wollrab & Diehl, 2015). In addition to this density‐dependent food uptake term, we consider a prey preference parameter *p*_*Z*_ $\in \left[0,1\right]$ which defines the preference level of *Z* for *P*_*E*_ (*p*_*Z*_) versus *F* (1−*p*_*Z*_). Accordingly, the total food uptake rate *a*_*Z*_ is divided between the two zooplankton prey species via multiplication by *p*_*Z*_ (1−*p*_*Z*_), to derive the effective food uptake rate for *P*_*E*_ (*F*), respectively. Correspondingly, *p*_*Z*_ < (>) 0.5 indicates a preference for *F* (*P*_*E*_) with an exclusive diet on *F* (*P*_*E*_) at the extreme values of *p*_*Z*_ = 0 (1) and *p*_*Z*_ = 0.5 indicating no preference. Consumed prey biomass is converted to zooplankton biomass by conversion efficiencies *e*_*P*_ and *e*_*F*_ for edible phytoplankton and fungi, respectively.

(1) $$\frac{\mathrm{dN}}{\mathrm{dt}}=q\left(N_{\max}-N\right)-\frac{\mu_{\max,E}N}{K+N}P_{E}-\frac{\mu_{\max,I}N}{K+N}P_{I,}$$

(2) $$\frac{dP_{E}}{\mathrm{dt}}=\frac{\mu_{\max,E}N}{K+N}P_{E}-\frac{p_{Z}\cdot a_{Z}\cdot P_{E}^{2}}{1+h_{P_{E}}\cdot p_{Z}\cdot a_{Z}\cdot P_{E}^{2}+h_{F}\cdot \left(1-p_{Z}\right)\cdot a_{Z}\cdot F^{2}}Z-qP_{E},$$

(3) $$\frac{dP_{I}}{\mathrm{dt}}=\frac{\mu_{\max,I}N}{K+N}P_{I}-\beta P_{I}F-qP_{I}\;,$$

(4) $$\frac{\mathrm{dF}}{\mathrm{dt}}=f_{F}\beta P_{I}F-\frac{\left(1-p_{Z}\right)\cdot a_{Z}\cdot F^{2}}{1+h_{P_{E}}\cdot p_{Z}\cdot a_{Z}\cdot P_{E}^{2}+h_{F}\cdot \left(1-p_{Z}\right)\cdot a_{Z}\cdot F^{2}}Z-\mathrm{qF},$$

(5) $$\frac{\mathrm{dZ}}{\mathrm{dt}}=\frac{\left(e_{p}\cdot p_{Z}\cdot a_{Z}{\cdot P}_{E}^{2}+e_{F}\cdot \left(1-p_{Z}\right)\cdot a_{Z}\cdot F^{2}\right)}{1+h_{P_{E}}\cdot p_{Z}\cdot a_{Z}\cdot P_{E}^{2}+h_{F}\cdot \left(1-p_{Z}\right)\cdot a_{Z}\cdot F^{2}}Z-\left(q+m_{Z}\right)Z.$$

(6) $$\frac{dp_{Z}}{\mathrm{dt}}=V\frac{\partial W_{Z}}{\partial p_{Z}}+B\left(p_{Z}\right).$$

For the (nonadaptive) fixed preference case, a representative for passive filter feeders like cladocerans, *p*_*Z*_ was assumed to be a constant parameter (Equations (1), (2), (3), (4), (5)), but not necessarily 0.5 (no preference), as even filter feeders have the ability to actively egest unfavorable prey (Uszko et al., 2015). For the adaptive preference case, a representative for active hunters like copepods (Ray et al., 2016), *p*_*Z*_ is not fixed, but itself a function of time (Equation 6). We used a fitness gradient approach to describe the adaptive preference dynamics (Abrams & Matsuda, 2004; Mougi & Iwasa, 2010; Yamamichi et al., 2019). For this case, the value of *p*_*Z*_ depends on the fitness gradient of *Z*, described by the effect of a change of *p*_*Z*_ on the net energy gain of zooplankton $\left(\frac{\partial W_{Z}}{\partial p_{Z}}\right)$, where $W_{Z}\left(t\right)=\frac{1}{Z}\cdot \frac{\mathrm{dZ}}{\mathrm{dt}}$ is the net growth of zooplankton. This fitness gradient is multiplied by the speed of adaptation *V*. This first term of Equation (6) describes the ability of *Z* to adapt its prey preference *p*_*Z*_ to optimize its own fitness (net energy gain). The second additive term of Equation (6) is a boundary function $B\left(p_{Z}\right)=\frac{C}{p_{Z}^{2}}-\frac{C}{{\left(1-p_{Z}\right)}^{2}}$ with the scaling constant *c*, which keeps the value of *p*_*Z*_ (*t*) within the range of [0,1] (Yamamichi et al., 2019).

Parameter values (Appendix S1: Table S1) and initial conditions (see section on model analysis) were taken from Miki et al. (2011), with the exception of the maximum growth rate of $P_{I}$ ($\mu_{\max,I}$). The maximum growth rate of *P*_*I*_ was chosen to be higher than in the original parameterization (increasing it from 50% to 95% of $\mu_{\max,E}$) to reduce the influence of differences in the physiological rates of edible versus inedible phytoplankton in this conceptual study. Such an assumption is well supported by empirical data, where differences in maximum growth rate between edible and inedible algae can be small (Burson et al., 2018).

### 2.2 Model analysis

For all simulations, we varied maximum nutrient availability *N*_max_ from 0.1 to 65 μgP L^−1^ in steps of 0.1, ranging from oligotrophic to eutrophic conditions (Carlson & Simpson, 1996). Equilibrium densities were examined along a grid of *p*_*Z*_ and *N*_max_ levels. We investigated the equilibrium dynamics of the study system for the case of (1) fixed food preference of *Z* for *P*_*E*_ versus *F*, covering the full range of possible preference levels within the interval of [0,1] and (2) adaptive food preference of Z.

*For the fixed preference case*, we numerically solved the system of Equations ((1), (2), (3), (4), (5)) for transects along *N*_max_ for fixed values of *p*_*Z*_. Transects were calculated for *p*_*Z*_ values from 0 to 1 in steps of 0.01 using a standard Runge–Kutta solver (*ode*45) in MATLAB R2018b (MathWorks). For each run, we integrated the system for 20,000 time units, starting from the initial conditions (μgP·L^−1^): *N* (0) = 8, *P*_*E*_ (0) = 0.472 × 10^−6^, *P*_I_ (0) = 8.68 × 10^−6^, *F* (0) = 2.4 × 10^−6^, and *Z* (0) = 0.21. For the forward calculations, the transects started at *N*_max_ = 0.1. The system dynamics were followed along with increasing values of *N*_max_ (in steps of 0.1) by using the solution reached at the end of the previous run to be the initial condition for the next run. As simulation output, for each run, the mean biomass of each state variable was calculated using the last 20% of total time steps. In case the system exhibited oscillatory dynamics, we also calculated the standard deviation of the mean. A species was considered to be extinct if its mean biomass over the last 20% of time steps was less than 0.0001 μgP L^−1^. In case of extinction at the end of the previous run, 0.0001 was added to the initial biomass of the corresponding population in the following run, allowing for reinvasion. The continuation was performed until reaching *N*_max_ = 65 μgP L^−1^. Following the same procedure, system dynamics were also continued for fixed values of *N*_max_ along increasing values of *p*_*Z*_. All transects were also followed in the opposite directions, that is, along decreasing values of *N*_max_ and *p*_*Z*_, results were identical to the forward calculations. Bifurcation analysis and specifically the detection of a Hopf bifurcation was performed using MatCont 7p2 (Dhooge et al., 2006), a software package used within MATLAB.

*For the adaptive preference case*, system dynamics for Equations ((1), (2), (3), (4), (5), (6)) were followed along with increasing *N*_max_, using the same procedure as for the fixed preference case, with starting values *N*_max_ = 0.1 and *p*_*Z*_ (0) = 0.5, increasing *N*_max_ by steps of 0.1. System dynamics of Equations ((1), (2), (3), (4), (5), (6)) tended to reach equilibrium dynamics faster than for the nonadaptive case (Equations (1), (2), (3), (4), (5)), therefore, for the adaptive preference case, system dynamics were only followed for 10,000 time units.

Furthermore, we calculated the *energy flow* for different prey preferences and nutrient levels. The energy flow can be assumed to be equivalent to the flow of the limiting nutrient (Rooney et al., 2006). Given that the biomasses in our model system are expressed in terms of the limiting nutrient, energy flow corresponds to the biomass flow along each trophic link. We evaluated two measures of energy flow along each trophic link: (1) net energy gain of the consumer *C* from its resource *R* (*g*_RC_) and (2) gross energy flow from resource *R* to consumer *C*. For example, the net energy gain of *Z* from consuming *P*_*E*_ is given by $g_{P_{E}Z}=\frac{e_{p}\cdot p_{Z}\cdot a_{Z}\cdot P_{E}^{2}\cdot Z}{1+h_{P_{E}}\cdot p_{Z}\cdot a_{Z}\cdot P_{E}^{2}+h_{F}\cdot \left(1-p_{Z}\right)\cdot a_{Z}\cdot F^{2}}$, whereas the gross energy flow along the $P_{E}-Z$ link is given by $\frac{g_{P_{E}Z}}{e_{P}}$. In the case of oscillatory dynamics, we used the mean biomass of all state variables (as described above) for the calculation of energy flow. The relative contribution of fungi to the net energy gain of zooplankton is calculated by $\frac{g_{\mathrm{FZ}}}{g_{P_{E}Z}+g_{\mathrm{FZ}}}\times 100$.

The transfer efficiency (*TE*) is calculated as the quotient of net energy gain of the consumer/parasite and net energy gain of the host/prey. To get transfer efficiency along the mycoloop ($TE_{P_{I}-F-Z}$), that is, the nutrient transfer from inedible host phytoplankton to zooplankton via consumption of parasitic fungi, the *TE* from host to fungi $TE_{P_{I}-F}\;\left[\%\right]=$ $\frac{g_{P_{I}F}}{g_{NP_{I}}}\times 100$ is multiplied by the *TE* from fungi to zooplankton $E_{F-Z}\;\left[\%\right]=$ $\frac{g_{\mathrm{FZ}}}{g_{P_{I}F}}\times 100$, that is, $E_{P_{I}-F-Z}$ $\left[\%\right]=TE_{P_{I}-F}\times TE_{F-Z}$.

## 3 RESULTS

### 3.1 Community patterns for the fixed preference case

**FIGURE 2:**
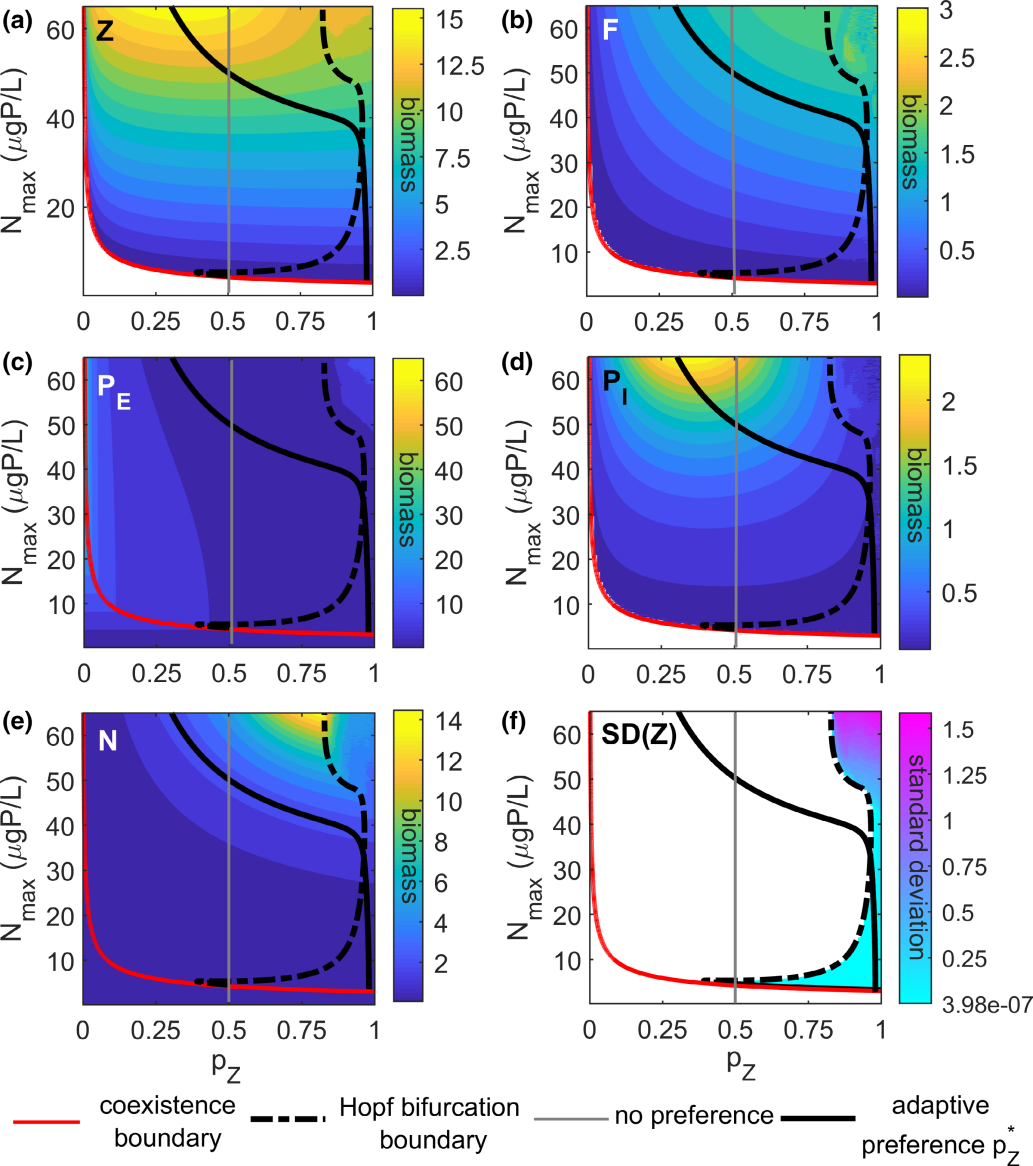
Effect of nutrient enrichment (*N*_max_) and prey preference (*p*_*Z*_) of zooplankton (*Z*) for edible phytoplankton (*P*_*E*_) and parasitic fungi (*F*), on species biomass and stability. Panels (a–e) illustrate the mean equilibrium biomass (μgP L^−1^) for (a) zooplankton *Z*, (b) parasitic fungi *F*, (c) edible phytoplankton *P*_*E*_, (d) inedible phytoplankton *P*_*I*_, and (e) freely available nutrient *N*, white areas indicate the extinction of the respective species. The dash‐dotted line (Hopf bifurcation boundary) separates the area of stable point equilibria (on the left) from the area with oscillatory behavior (on the right). The optimal preference ($p_{Z}^{*}$) is only illustrated within the area of coexistence (*N*_max_ > 2.7 μgP L^−1^). Panel (f) shows the standard deviation of the mean biomass of zooplankton for regions with oscillatory behavior.

Along the enrichment gradient (*N*_max_), the superior resource competitor (edible phytoplankton) can establish at the lowest enrichment level (*N*_max_ > 0.055 μgP L^−1^), independent of the preference value. The invasion threshold for zooplankton depends on the available prey biomass and the preference value for edible phytoplankton, with the enrichment level for successful invasion increasing with decreasing preference for the phytoplankton (*N*_max_ > 2.9 μgP L^−1^). For enrichment levels slightly above the invasion threshold for zooplankton, the inedible phytoplankton and finally the parasitic fungi can successfully invade. The invasion boundaries for zooplankton, inedible phytoplankton, and fungi are very close to each other, nearly overlapping, therefore, only the coexistence boundary is indicated in Figure 2 (red curve). Coexistence is not possible for too low enrichment levels (*N*_max_ < 3.05 μgP L^−1^) and too strong preferences for fungi (*p*_*Z*_ close to zero).

Within the coexistence area, the mean biomass of all food web compartments along the mycoloop increases with nutrient enrichment (Figure 2a,b,d,e), whereas, it decreases for edible phytoplankton (Figure 2c). The community is dominated by either edible phytoplankton (at low nutrient availability) or zooplankton (at high nutrient availability, apart from regions with an almost exclusive preference for fungi; see Appendix S2, Figure S2.1).

The response of zooplankton along the preference gradient differs for low versus high enrichment levels (Figure 2a). For low enrichment levels, zooplankton increases with an increasing preference for edible phytoplankton. For high enrichment levels, zooplankton shows a hump‐shaped relationship reaching the highest biomass at $\;p_{Z}\approx 0.3$. With an increasing preference for edible phytoplankton, the phytoplankton decreases due to stronger top‐down pressure through zooplankton (Figure 2c), whereas the biomass of fungi increases (Figure 2b). The abundance of the fungal host (inedible phytoplankton) shows a hump‐shaped relationship with respect to the preference, peaking at $p_{Z}\approx 0.3$, where its parasite is the preferred prey (Figure 2d). For high enrichment levels, the area with the highest inedible phytoplankton biomass overlaps with the region of maximum biomass of zooplankton (Figure 2a,d), indicating a strong top‐down control on both prey species, releasing the phytoplankton host from infection through fungi and from the nutrient competition with edible phytoplankton.

At preference values close to one (i.e., strong preference for edible phytoplankton), the system exhibits oscillatory dynamics. This region extends toward lower preference levels with nutrient enrichment (area to the right of the black dashed line in Figure 2). The oscillatory dynamics are characterized by small amplitude cycles for low enrichment and a pronounced increase of cycle amplitudes at high enrichment levels (Figure 2f).

In the area with stable point equilibria (area to the left of the black dashed line in Figure 2), all compartments reach their maximum biomass at the highest investigated enrichment level (Figure 2a–e). Only in the absence of zooplankton (edible phytoplankton‐only state), for preference values close to zero, the edible phytoplankton increase with enrichment (area to the left of the red line in Figure 2c). The maximum fungal biomass and freely available nutrient levels are observed at high preference values for edible phytoplankton ($p_{Z}\approx$ 0.8; Figure 2b,e).

**FIGURE 3:**
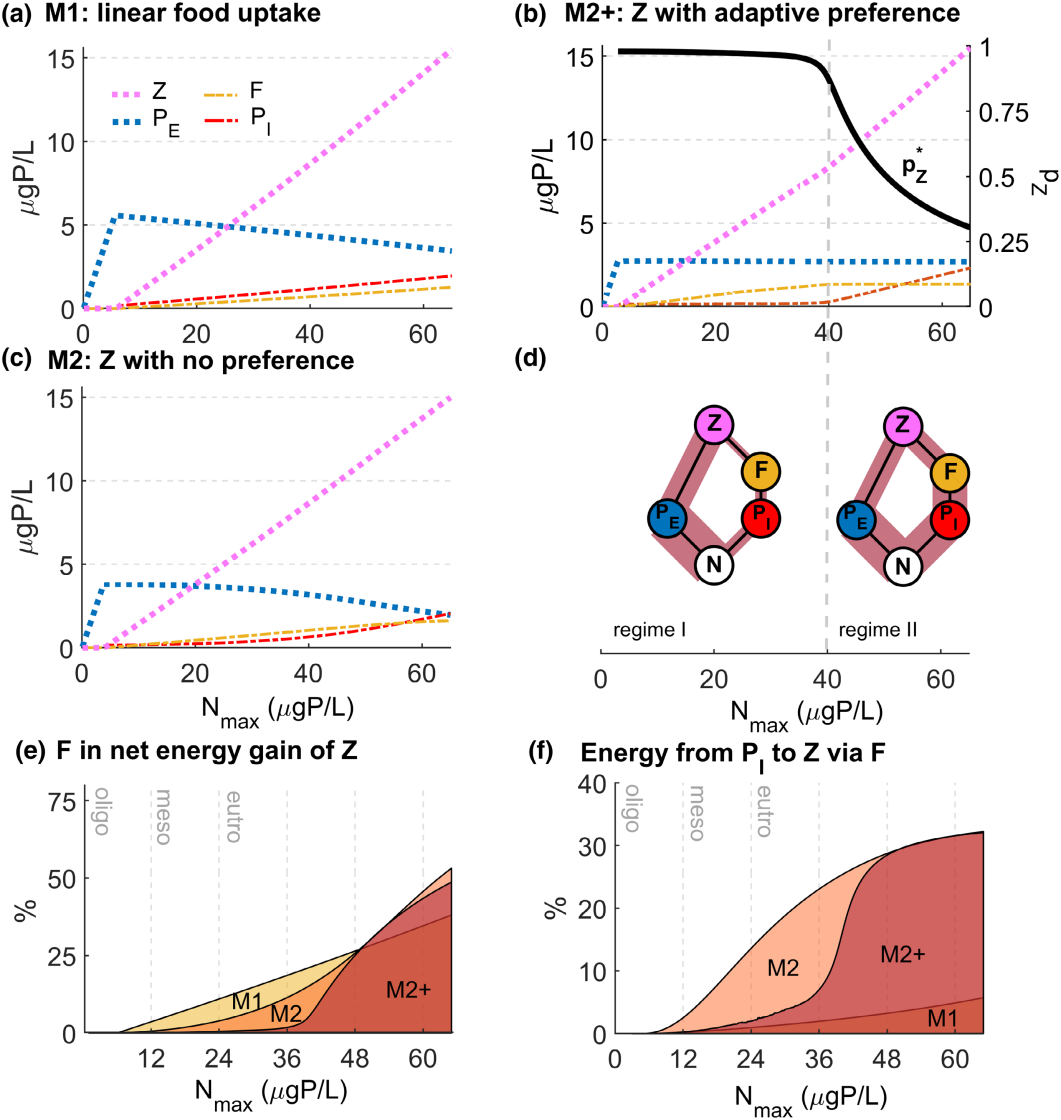
Changes in the mean equilibrium biomass of all food web compartments with increasing *N*_max_ under the assumption of (a) linear food uptake terms (Miki et al., 2011) (M1), (b, c) saturating food uptake terms for (b) nonadaptive zooplankton with no prey preference (*p*_*Z*_ = 0.5) (M2), (c) adaptive zooplankton with adaptive prey preference (*p*_*Z*_ (*t*)) (M2+). In panel (d), the distribution of energy flow in the food web is illustrated for Regime I and Regime II of M2+. The relative energy flow along each interaction link is illustrated by the width of the brown shaded area for Regime I (at *N*_max_ = 25 μgP L^−1^) and Regime II (at *N*_max_ = 65 μgP L^−1^ and $p_{Z}^{*}$ = 0.3034). Panels (e, f) show a comparison of M1, M2, and M2+ for (e) the relative contribution of fungi to the net energy gain of zooplankton and (f) transfer efficiency along the mycoloop.

In comparison with the assumption of linear food uptake rates (Miki et al., 2011), there is no qualitative change in the biomass response pattern along the nutrient gradient under the assumption of nonlinear food uptake rates, as illustrated for *p*_*Z*_ = 0.5 (Figure 3a,b). However, while the host phytoplankton is predicted to reach higher biomass compared with its parasite throughout the nutrient gradient for the linear case, under the assumption of saturating food uptake terms, the phytoplankton host only dominates at the highest nutrient levels (see Appendix S2, Figure S2.2). Furthermore, compared with the linear case, zooplankton can invade at lower prey abundance and lower *N*_max_ (*N*_max_ = 5.6, 3.8, and 2.7 [μgP L^−1^] for linear, nonlinear with no preference, and nonlinear with adaptive preference case, respectively), so edible phytoplankton cannot reach as high biomass levels and decreases more steeply along the nutrient gradient compared with the linear case (Figure 3a,b).

### 3.2 Community patterns for the adaptive preference case

**FIGURE 4:**
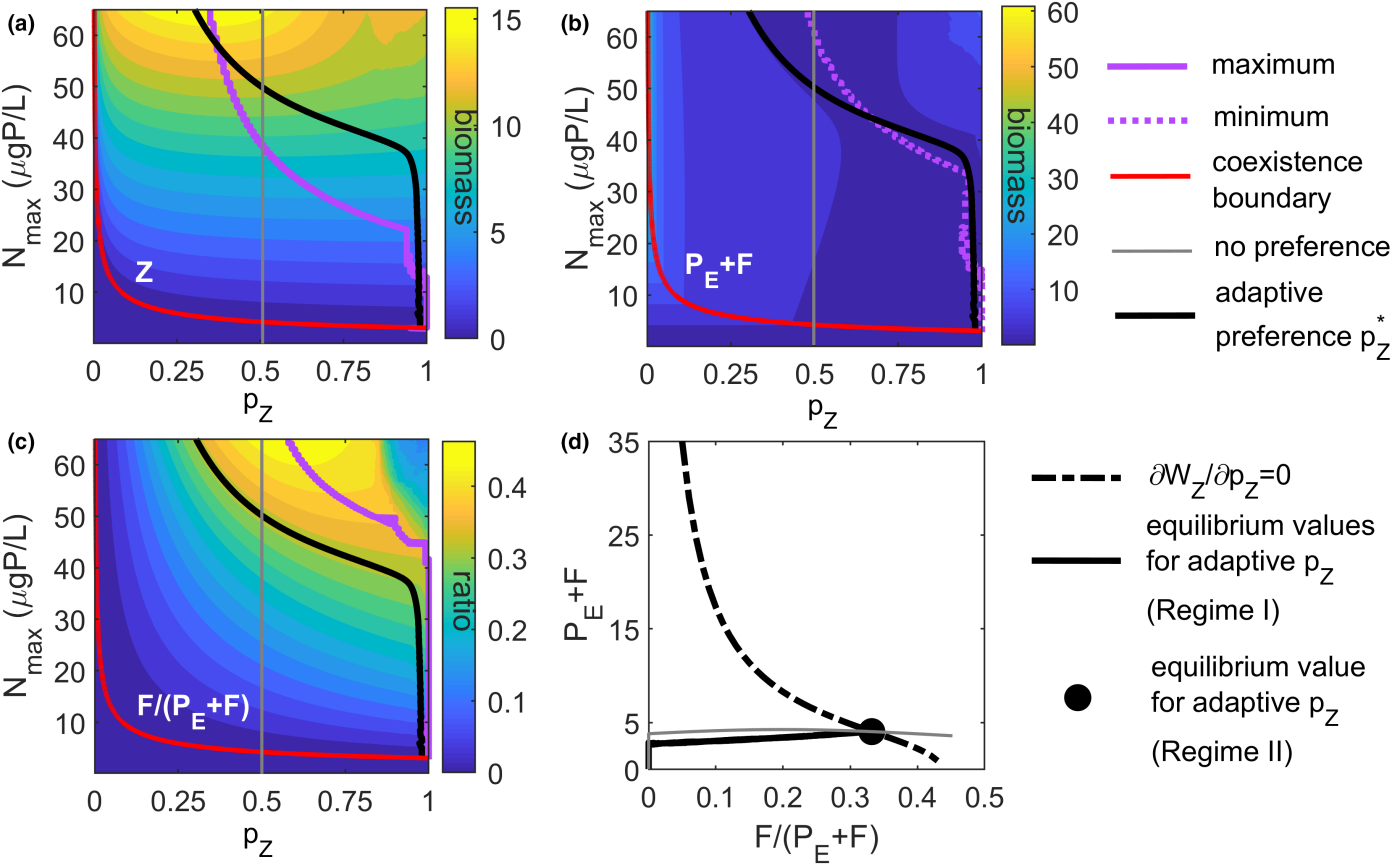
Illustration of optimal prey preference values ($p_{Z}^{*}$) for the case of adaptive zooplankton (black solid line) in comparison with maximum (a) zooplankton biomass, (c) relative contribution of parasite (*F*) to total prey, indicated by a pink solid line, and (b) minimum total prey biomass (*P*_*E*_ + *F*) indicated by a pink dotted line. Panel (d) illustrates the optimal prey availability following the optimal fitness strategy $\frac{\partial w_{Z}}{\partial p_{Z}}=0$ (dash‐dotted line) and its dependence on the relative contribution of fungi to total prey biomass. Solid lines indicate the calculated values for the adaptive preference case with increasing *N*_max_ under Regime I. The intersection point of calculated and optimal values is indicated by a point and represents the equilibrium value reached under Regime II.

Following the optimal preference values $p_{Z}^{*}$ (average equilibrium value of *p*_Z_ (*t*) in case of oscillatory dynamics) which are reached along with nutrient enrichment for the adaptive preference case (Equations (1), (2), (3), (4), (5), (6)), two different regimes can be distinguished for low versus high enrichment levels (Figures 2 and 3c). In Regime I (*N*_max_ < 40 μgP L^−1^), edible phytoplankton clearly dominates the total available prey for zooplankton and correspondingly, the optimal preference is almost exclusive for edible phytoplankton (*p*_*Z*_ close to 1; Figures 3c and 4c). In Regime II (*N*_max_ > 40 μgP L^−1^), with biomass of fungi and its relative contribution to total prey biomass reaching a critical threshold (Figure 4d), optimal preference exhibits a pronounced shift toward a preference for fungi with further enrichment, even though edible phytoplankton still dominates the total available prey (Figure 3c). In Regime I, the qualitative pattern of the community response to nutrient enrichment is identical to the fixed preference case (Figure 3b,c), with edible phytoplankton slightly decreasing. In Regime II, the freely available nutrient (*N*) and mean biomasses of both zooplankton prey remain constant (Figures 2e, 3c and 4d), keeping the relative contribution of fungi to total available prey biomass at 33% (Figures 4c and 3d). Only zooplankton and inedible phytoplankton (host) increase with further nutrient enrichment (Figure 3c). Zooplankton biomass increases more steeply with nutrient enrichment compared with Regime I (Figure 3c) and reaches higher maximum biomass compared with the case without prey preference (*p*_*Z*_ = 0.5; Figure 4a). The equilibrium dynamics exhibit the same stable versus oscillatory behavior as the fixed preference case for the respective parameter combination on the *p*_*Z*_−*N*_max_ plane.

It is notable that the adaptive preference values neither follow maximum zooplankton biomass (Figure 4a) nor values with the highest top‐down control and, therefore, the lowest total prey biomass (Figure 4b). They also do not follow the highest biomass values of fungi, although being the more profitable prey for zooplankton (greater conversion efficiency of fungi than edible phytoplankton; Figure 4c). So, what governs the optimal preference value along the enrichment gradient and how is this related to total and relative prey densities?

Analyzing the equilibrium condition for the fitness gradient term of Equation (6) ($\frac{\partial W_{Z}}{\partial p_{Z}}=0$), which optimizes zooplankton net growth ($W_{Z}=\frac{1}{Z}\cdot \frac{\mathrm{dZ}}{\mathrm{dt}})$, reveals a negative correlation between the relative contribution of fungi to total prey biomass (*F*/(*P*_*E*_ + *F*)) and total prey biomass (*P*_*E*_ + *F*; Figure 4d). Comparing these optimal prey availabilities (black dash‐dotted line in Figure 4d) with the simulated equilibrium values for the mycoloop food web (Equations (1), (2), (3), (4), (5), (6); black solid line in Figure 4d) reveals that the optimal value of the fitness gradient term cannot be reached before prey composition along the enrichment gradient reaches the optimal prey availability (black dot in Figure 4d). Once reached, total and relative prey values are preserved at these values with further enrichment, while the optimal preference value keeps decreasing (increasing preference for fungi) with further nutrient enrichment (for further details see Appendix S3).

### 3.3 Energetic role of the mycoloop

In the investigated mycoloop food web, there are two alternative energy pathways to zooplankton: one from edible nonhost phytoplankton directly and the other from parasitic fungi emerging from inedible host phytoplankton (mycoloop Figure 1).

A comparison between three sets of assumptions, (M1) linear food uptake rates for all species with zooplankton without prey preference (*p*_*Z*_ = 0.5; as in Miki et al., 2011), (M2) saturating food uptake rates for phytoplankton and zooplankton without prey preference, and (M2+) in addition to M2, zooplankton with adaptive prey preference (Figure 3), shows that qualitative predictions, but not the quantitative predictions on the dominance of energy flow between both pathways is independent of the zooplankton feeding strategy. A strong dominance of energy flow along the direct phytoplankton pathway is predicted at low nutrient availabilities. Increasing importance of energy flow along the mycoloop is predicted with nutrient enrichment (Figure 3e,f). This reflects the increase in fungi biomass and the decrease in edible phytoplankton biomass along the nutrient gradient (Figure 3a–c). For the adaptive preference case, the increasing importance of the mycoloop along the nutrient gradient is much more pronounced, with almost exclusive preference of zooplankton for edible phytoplankton in Regime I to equal importance of energy flow between both energy pathways in Regime II (Figure 3d–f). Energy flow along the mycoloop would even dominate for nutrient enrichment levels beyond the investigated values (see Appendix S4).

With respect to the quantitative predictions, the three model assumptions (M1, M2, and M2+) differ quite significantly in their predictions of the transfer of energy from host to fungi along the nutrient gradient (Figure 3e,f). At low nutrient availability, predictions on the net energy gain of zooplankton from fungi are the highest under the assumption of linear food uptake terms (Figure 3e). However, at high nutrients, the assumption of saturating food uptake terms leads to much higher predictions on the net energy gain of zooplankton from fungi relative to edible phytoplankton, reaching up to 50%–55%, while the net energy gain stays well below 40% for the linear case (Figure 3e). The difference in predictions is even more pronounced for the transfer efficiency along the mycoloop, which reaches 30% under the assumption of saturating functional responses, while it remains below 5% under the assumption of linear food uptake rates (Figure 3f). For the two scenarios with saturating functional responses (M2, M2+), at lower nutrient availabilities, the transfer efficiency is the highest for the case of fixed preference (*p*_*Z*_ = 0.5, no preference), however, the adaptive preference case reaches similarly high values under high nutrient availabilities (Regime II; Figure 3f).

## 4 DISCUSSION

Our model results highlight the critical role of parasite‐mediated energy pathways for community response along a nutrient gradient. It furthermore highlights the potential importance of such pathways for energy flow to higher trophic levels, exemplified by the mycoloop web, and how this is modulated by consumer feeding strategies (active adaptive versus passive nonadaptive). This study pays specific attention to energy pathways created by the direct consumption of parasites emerging from otherwise inedible host species.

### 4.1 Regime shift from low to high contribution of fungi in zooplankton diet

Although we observe a smooth increase in energy flow through the mycoloop pathway with nutrient enrichment for a nonadaptive consumer, for an adaptive consumer, our results suggest an abrupt shift from the dominance of energy flow through the direct edible (nonhost) phytoplankton–zooplankton pathway at low nutrient levels (Regime I) to equal dominance of energy flow along both (edible phytoplankton and parasite) pathways at high nutrient levels (Regime II). Our study specifically indicates that parasitic fungi can contribute 50% or more to the diet of zooplankton in nutrient‐rich environments with the dominance of inedible phytoplankton. This clearly exceeds predictions under the assumption of linear feeding interactions (Miki et al., 2011) and is supported by empirical observations showing that fungal zoospores can contribute 50%–60% to the zooplankton diet during phytoplankton blooms dominated by inedible species (Rasconi et al., 2014). Our finding extends on previous theoretical and empirical work, highlighting how energy flow along the mycoloop influences plankton community dynamics and its dependence on zooplankton feeding strategy.

### 4.2 Optimal prey availability for zooplankton depends on community feedback

A notable result is that the reachability of an optimal prey preference might be limited by the food web response due to a trade‐off between total prey biomass and relative contribution of the more profitable prey (fungi) to total prey. In contrast to indications from previous studies on optimal foraging on multiple prey (Visser & Fiksen, 2013), our results show that optimality might not be reached before a critical threshold of relative and total prey availability is reached, which itself is constrained by the community response along the nutrient gradient. Furthermore, the comparison of biomass patterns for the fixed and the adaptive preference case shows that the optimization of net energy gain does not necessarily maximize consumer biomass. These results suggest that the codependence of relative and total prey availability and the negative correlation between alternative prey species effectively keeps the adaptive preference function from maximizing consumer biomass. It would be interesting to look at the general relevance of this finding for adaptive predation in more complex communities.

### 4.3 Contributions to food web theory

This study also adds new aspects to the importance of food web structure for food web dynamics (Drossel et al., 2001; O'Gorman et al., 2010) and how this is modulated by species‐specific rates (Gibert & DeLong, 2017) and trait adaptation (Cattin et al., 2004), which can also stem from behavioral responses. The community response pattern with an increase of all species along the mycoloop but a decrease of edible phytoplankton with increasing nutrient availability (nonadaptive case and Regime I) follows the dynamics predicted for food webs consisting of one chain of even and one chain of odd length, which are connected via a shared resource and a shared predator (Wollrab et al., 2012). Similar to predictions from classic food web theory on predator‐mediated coexistence between competing prey species (Holt et al., 1994; Leibold, 1996), we also observe a shift from the dominance of exploitative to apparent competition for the mycoloop web, reflected by the initial dominance and successive decrease (increase) of the superior (inferior) resource competitor with nutrient enrichment. While inedible phytoplankton would profit from enrichment even in the absence of the mycoloop (no loss due to feeding/parasitism), in its presence, zooplankton gains additional energy that results in increased predation pressure on edible phytoplankton, its competitor. However, the observed match with general topological features from classic trophic interaction food webs might in part stem from the implementation of the parasite–host as a predator–prey interaction, although with a much lower production rate of chytrids per unit host biomass compared with a classic predator (see, for example, Wollrab et al., 2015).

Furthermore, a comparison between dynamic properties of the structurally equivalent plankton food web (Stibor et al., 2004; Thingstad & Sakshaug, 1990; Wollrab & Diehl, 2015), where ciliates are structurally at the same position as parasitic fungi in the mycoloop food web, provides new insight into the occurrence of abrupt shifts in community response along a nutrient gradient. For both webs, the occurrence of a regime shift in community response is critically related to the assumption of an adaptive feeding strategy for the consumer (see Appendix S5). This in combination with the topologically constrained community response, where ciliates/fungi increase with nutrient enrichment while the alternative prey decreases, leads to a disproportional (abrupt) shift in prey preference for ciliates/fungi along the nutrient gradient (Wollrab et al., 2020; Wollrab & Diehl, 2015). Notably, in both cases, this abrupt shift in prey preference creates a bottleneck in energy flow and leads to a drastic shift in community responses to further enrichment, which is absent if assuming a nonadaptive consumer (for further details, see Appendix S5). This finding reveals the critical interplay of structural features, functional response type, and production rates for the occurrence of abrupt shifts in community composition along a nutrient gradient (see details in Appendix S5).

### 4.4 System stability

Our analysis of the mycoloop food web also supports the potentially stabilizing role of parasites for system dynamics (Lafferty et al., 2006; Rogawa et al., 2018). The growth/infection rate of phytoplankton versus parasite determines the amount of energy (biomass) that can be produced per unit of time. Given the large difference in phytoplankton growth versus fungal infectivity rate in our study system, the path from edible phytoplankton to zooplankton can be characterized as a fast energy pathway, whereas the path from fungi to zooplankton can be considered a slow energy pathway. Hence, with an increasing preference for parasitic fungi, the slow energy channel stabilizes the oscillatory dynamics of the fast energy channel (Blanchard et al., 2011; Gellner & McCann, 2016; Rooney et al., 2006, see also Appendix S3, Figure S3.3). However, we have to caution that the observed stabilizing role of the mycoloop might partly be due to the simplified representation of the host–parasite interaction, which ignores the time lag between parasite infection and zoospore emergence. A differentiation between susceptible and infected hosts (SI model) might to some extent counteract the stabilizing features.

### 4.5 Parasites under global warming and eutrophication

Given the empirical counterpart of our modeled system, our results are of high relevance in light of global warming and eutrophication, not only increasing the risk of blooms dominated by cyanobacteria or other inedible phytoplankton species (Davis et al., 2009; Paltsev & Creed, 2022) but also influencing the prevalence of parasitic infections (Gsell et al., 2013; Harvell et al., 2002; Ibelings et al., 2011), which for temperate regions are expected to rise with increasing temperature (Cohen et al., 2020). Based on direct phyto–zooplankton interactions, a decline in zooplankton would be expected with the increasing dominance of inedible phytoplankton (Lampert, 1987). However, our results suggest that this might be counteracted if parasites form an alternative food source for zooplankton (Agha et al., 2018; Frenken, 2018; Kagami et al., 2007). On the other hand, in tropical regions where the prevalence of infection is predicted to decline with increasing temperature (Cohen et al., 2020), the importance of the mycoloop for zooplankton might decrease.

## 5 CONCLUSIONS

Extending on an existing theory (Miki et al., 2011) by taking nonlinear feeding interactions and different zooplankton feeding strategies into account, our model analysis provides a more realistic prediction of the importance of energy flow along the mycoloop for major feeding guilds, that is, nonadaptive filter feeders like cladocerans versus adaptive active hunters like raptorial copepods. Our study highlights the potentially crucial role of dominant zooplankton feeding guilds in the community response to environmental change. More specifically, parasite‐mediated energy pathways from inedible hosts to nonhost consumers via consumption of propagules or ectoparasites might form a crucial link for community response to environmental change and predictions on corresponding changes in trophic transfer efficiency. Additionally, the obtained limitations on optimal prey choice in the context of food web topology and corresponding community feedback have implications far beyond the investigated study system.

## AUTHOR CONTRIBUTIONS

**Patch Thongthaisong:** Conceptualization (equal); formal analysis (equal); investigation (equal); methodology (equal); visualization (lead); writing – original draft (lead); writing – review and editing (equal). **Minoru Kasada:** Formal analysis (supporting); supervision (supporting); visualization (supporting); writing – review and editing (supporting). **Hans‐Peter Grossart:** Conceptualization (equal); funding acquisition (lead); methodology (supporting); supervision (equal); writing – review and editing (equal). **Sabine Wollrab:** Conceptualization (lead); formal analysis (equal); funding acquisition (lead); investigation (equal); methodology (equal); project administration (lead); resources (lead); software (lead); supervision (lead); validation (lead); visualization (equal); writing – review and editing (equal).

## FUNDING INFORMATION

This project was funded by the German Research Foundation (DFG) Priority Program 1704: DynaTrait (WO 2273/1‐1) and (GR 1540/30‐1). MK received funding from the Grant‐in‐Aid for JSPS Fellows (MK 19J00864). The work was partly funded by the German Federal Ministry of Education and Research BMBF within the Collaborative Project “Bridging in Biodiversity Science – BIBS” (grant number 01LC1501). All responsibility for the content of this publication is assumed by the authors.

## Supporting information


Data S1:

## Data Availability

No empirical data was used in this research.
