# Supplementary material for: Critical role of parasite‐mediated energy pathway on community response to nutrient enrichment
Source: Ecol Evol. 2022 Dec 13;12(12):e9622. doi: 10.1002/ece3.9622 (PMC9748242; doi:10.1002/ece3.9622)
Supplement: Supplementary file 1 — Data S1: [file ECE3-12-e9622-s001.docx]

Supplemental material on ‘**Critical role of parasite-mediated trophic interactions for energy flow and community dynamics’**

**S1. Variables and parameterization**

**Table S1** Variables and parameters

| Variables  /parameters | Values | Units | Description |
| --- | --- | --- | --- |
| $N$ | variable | $\mu gP\cdot L^{-1}$ | Dissolved nutrient density |
| $P_{E}$ | variable | $\mu gP\cdot L^{-1}$ | Biomass of edible phytoplankton |
| $P_{I}$ | variable | $\mu gP\cdot L^{-1}$ | Biomass of inedible phytoplankton |
| $F$ | variable | $\mu gP\cdot L^{-1}$ | Biomass of parasitic fungi |
| $Z$ | variable | $\mu gP\cdot L^{-1}$ | Biomass of zooplankton |
| $N_{max}$ | $[0.1,65]$ | $\mu gP\cdot L^{-1}$ | Maximum nutrient density |
| $q$ | $0.05$ | $day^{-1}$ | Dilution rate |
| $m_{Z}$ | $0.01$ | $day^{-1}$ | Mortality rate of $Z$ |
| $\mu_{max,E}$ | $0.9$ | $day^{-1}$ | Maximum growth rate of $P_{E}$ |
| $\mu_{max,I}$ | $0.855$ | $day^{-1}$ | Maximum growth rate of $P_{I}$ |
| $K$ | $0.929$ | $\left( \mu gP\cdot L \right)^{-1}$ | Half-saturation constant of $P_{E}$ and $P_{I}$ |
| $a_{Z}$ | $0.0720$ | ${L\cdot\left( \mu gP\cdot day \right)}^{-1}$ | Food uptake rate of Z for both prey species |
| $h_{P_{E}}$ | $3.09$ | $day$ | Handling time of $Z$ for $P_{E}$ |
| $h_{F}$ | $0.6077$ | $day$ | Handling time of $Z$ for $F$ |
| $e_{P}$ | $0.3$ | dimensionless | Conversion efficiency of $Z$ for $P_{E}$ |
| $e_{F}$ | $0.5$ | dimensionless | Conversion efficiency of $Z$ for $F$ |
| $\beta$ | $0.416$ | ${L\cdot\left( \mu gP\cdot day \right)}^{-1}$ | Infection rate of $F$ on $P_{I}$ |
| $f_{F}$ | $0.75$ | dimensionless | Conversion efficiency of $F$ for $P_{I}$ |
| $p_{z}$ | $[0,1]$ | $[0,1]$ variable | Feeding preference of $Z$ for $P_{E}$ |
| $V$ | $0.1$ | dimensionless | Speed of adaptation |
| $c$ | $0.000001$ | $day^{-1}$ | Scaling constant for boundary function |

**S2. Dominant species along prey preference and nutrient gradient**


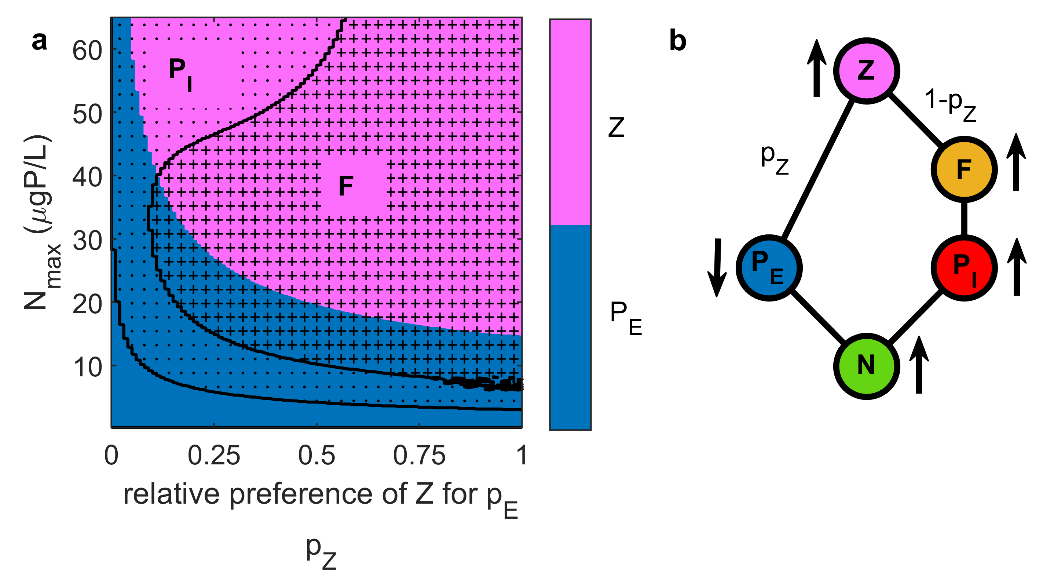


**Figure S2.1** *Community composition and equilibrium biomass dependent on maximum nutrient availability (*$N_{max}$*) and prey preference (*$p_{Z}$*) of zooplankton (*$Z$*) for the fixed preference case. In panel (a) areas of dominance of edible phytoplankton* $P_{E}$ *or* $Z$ *are indicated by blue or pink shading, respectively. Areas where the parasite* $F$ *dominates over its host* $P_{I}$ *are indicated by ‘+’, areas with dominance of* $P_{I}$ *over* $F$ *are indicated by ‘∙’. No coexistence is possible in the non-shaded blue area. Panel (b) illustrates the biomass response pattern of each food web compartment for the fixed preference case with increasing* $N_{max}$*. Thereby, circles represent the different species of the food web, connections between circles indicate feeding interactions and arrows next to the circles indicate the corresponding change in average biomass of the respective compartment with increasing* $N_{max}$*.*

Looking at the community composition over the investigated range of enrichment and preference levels, the community is either dominated by edible phytoplankton or zooplankton (Fig. S2.1a). For low to intermediate nutrient enrichment levels, edible phytoplankton dominates over the full preference range, but this range decreases with increasing enrichment until, at the highest enrichment levels, the dominance of edible phytoplankton is limited to preference values close to one, where only edible phytoplankton is able to persist. Correspondingly, zooplankton dominates for intermediate to high enrichment levels with dominance of zooplankton extending towards increasing preference for fungi with nutrient enrichment. The area, where inedible phytoplankton reaches its maximum biomass, overlaps with the area where it dominates over its parasite (Fig. S2.1a) and even over its competitor (Fig. S2.2). The maximum of fungal biomass and freely available nutrients are observed at high preference values for edible phytoplankton ($p_{Z}\approx$ 0.8).


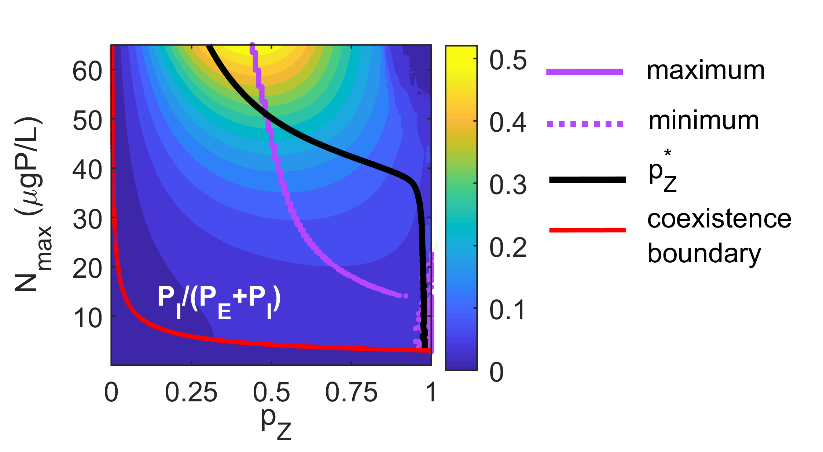


**Figure S2.2** *Contribution of inedible phytoplankton (*$P_{I}$*), which is the host of the fungi (*$F$*), to total phytoplankton biomass (P_E_ + P_I_).*

**S3. Optimal prey availability**

According to the chosen fitness gradient approach, assuming that the boundary function is negligibly small, which is true for optimal preference values not too close to its extreme values, adaptive preference optimizes the net-growth of zooplankton $W_{Z}=\frac{1}{Z}\cdot\frac{dZ}{dt}$ if the first term of Eq. 6 is zero, i.e.

| $\frac{\partial W_{Z}}{\partial p_{Z}}=0$. | (S3.1a) |
| --- | --- |

The solution to Eq. S3.1a can be re-arranged to indicate the relation between total and relative prey densities:

| $\left( P_{E}+F \right)^{2}=\frac{\left( \frac{F}{\left( P_{E}+F \right)} \right)^{2}\cdot e_{F}-\left( 1-\frac{F}{\left( P_{E}+F \right)} \right)^{2}\cdot e_{P}}{\left( e_{P}h_{F}-e_{F}h_{P_{E}} \right){\cdot a}_{Z}\cdot\left( \frac{F}{\left( P_{E}+F \right)} \right)^{2}}$ . | (S3.1b) |
| --- | --- |

Eq. S3.1b reveals that the equilibrium condition of the fitness gradient is independent of $p_{Z}$. Furthermore, it discloses a negative correlation between the relative contribution of fungi to total prey biomass$(F/(P_{E}+F))$ and total prey biomass ($P_{E}+F)$. The corresponding relationship between optimal relative fungi availability ranging from 0 to 1 and optimal total prey biomass is illustrated by the dash-dotted line in Fig. 3d (main manuscript). Please note that for values of relative fungal availability above $0.45$ the corresponding total prey biomass would get negative, setting an upper boundary on the relative contribution of fungi to the total prey biomass that could possibly be reached by an adaptive preference strategy.

Eq. S3.1a can also be re-arranged to give the relationship between $W_{Z}^{+}$, the growth term of zooplankton, and total and relative prey availability:

| $W_{Z}^{+}=\frac{total^{2}\left( e_{p}\cdot p_{Z}\cdot a_{Z}\cdot\left( 1-R_{F} \right)^{2}+e_{F}\cdot\left( 1-p_{Z} \right)\cdot a_{Z}\cdot R_{F}^{2} \right)}{1+total^{2}(h_{P_{S}}\cdot p_{Z}\cdot a_{Z}\cdot\left( 1-R_{F} \right)^{2}+h_{F}\cdot\left( 1-p_{Z} \right)\cdot a_{Z}\cdot R_{F}^{2})}$, | (S3.2) |
| --- | --- |

where $total=P_{E}+F$ and $R_{F}=F/total$. Under equilibrium condition, for each population gain and loss of energy needs to be balanced. Correspondingly, for zooplankton $\frac{dZ}{dt}=0$ (Eq. 5), this is fulfilled if the food uptake term $W_{Z}^{+}$(i.e. energy gain), where $W_{Z}^{+}=\frac{\left( e_{p}\cdot p_{Z}\cdot a_{Z}{\cdot P}_{E}^{2}+e_{F}\cdot\left( 1-p_{Z} \right)\cdot a_{Z}\cdot F^{2} \right)}{1+h_{P_{S}}\cdot p_{Z}\cdot a_{Z}\cdot P_{E}^{2}+h_{F}\cdot\left( 1-p_{Z} \right)\cdot a_{Z}\cdot F^{2}}$, equals the total loss term $W_{Z}^{-}$ (i.e. energy loss) ($W_{Z}^{-}=q+m_{Z}=0.06$). Similar to Fig. 3d, the dash-dotted line in Fig. S3.1 illustrates optimal prey availability, but now with respect to the relation between $W_{Z}^{+}$ and relative contribution of fungi to total prey biomass. The black solid line in Fig. S3.1 represents the calculated equilibrium values for $W_{Z}^{+}$ vs. relative contribution of fungi to total prey biomass for the adaptive preference case with increasing $N_{max}$. Accordingly, along this line the equilibrium condition of Eq. 5 ($W_{Z}^{+}=W_{Z}^{-})$ is fulfilled. However, only at the intersection between dash-dotted and solid line, indicated by the black dot, optimal prey availability can be reached under equilibrium conditions for Eqs. 1-6.

It should be noted that the assumption of a negligible influence of the boundary function on the prey preference value is not fulfilled for low $N_{max}$ in Regime I, where $P_{E}$ is strongly dominating the total prey biomass and the optimal prey preference therefore would be $p_{Z}^{*}=1$, reducing the zooplankton diet to a single prey. In this regime the boundary function $B(p_{Z})$keeps the adaptive preference from reaching the optimal value of one (see Fig. S3.2).

**
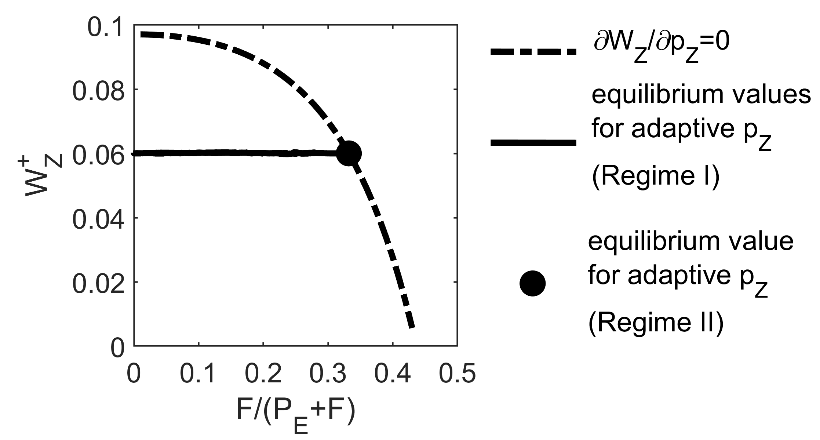
**

***Figure S3.1*** *Illustration of the optimal values according to Eq. S3.2 (derived from Eq. S3.1a,* $\frac{\partial w_{Z}}{\partial p_{Z}}=0$*, dash-dotted line) for optimal energy gain by zooplankton dependent on relative contribution of fungi to total prey biomass. Solid lines indicate the simulated values for the adaptive preference case with increasing N_max_ under Regime I. The intersection point of simulated and optimal values at the intersection point of the dash-dotted and solid line is indicated by a point and represents the equilibrium value reached under Regime II.*


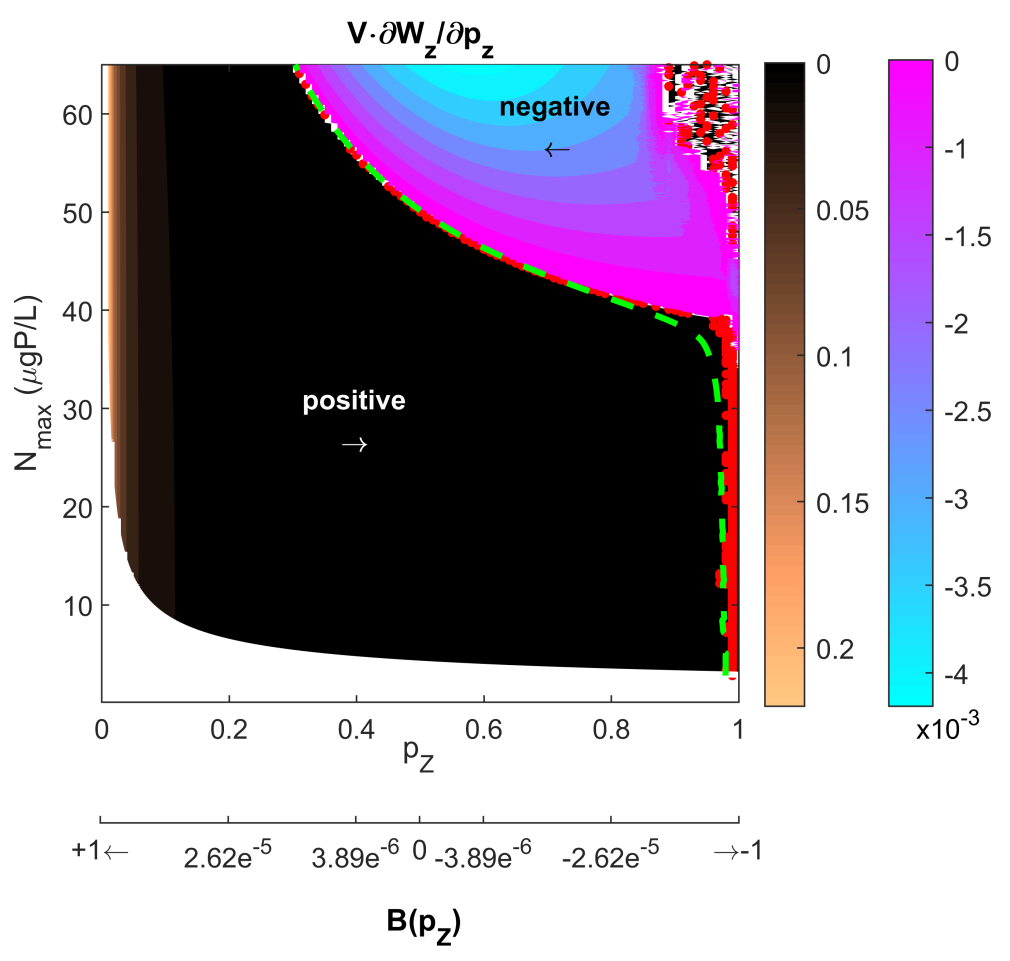


***Figure S3.2*** *Contour plot of the fitness gradient of zooplankton* $\frac{\partial W_{Z}}{\partial p_{Z}}$*, multiplied by the speed of adaptation V, on the* $p_{z}-N_{max}$ *plane. Decreasingly positive values of* $V\frac{\partial W_{Z}}{\partial p_{Z}}$ *are indicated by contours ranging from cream to black, for negative values decreasing absolute values are indicated by contours ranging from blue to pink. The second x-axis indicates the corresponding values of the boundary function* $B(p_{Z})$*. Arrows on the* $p_{z}-N_{max}$ *plane indicate the direction of change of the adaptive preference* $p_{Z}(t)$ *from any given value in the respective area. Red dots indicate minimum values of* $|V\frac{\partial W_{Z}}{\partial p_{Z}}|$ *for each Nmax. The green dashed line indicates the optimal preference* $p_{Z}^{*}$ *as calculated from Eqs 1-6.*


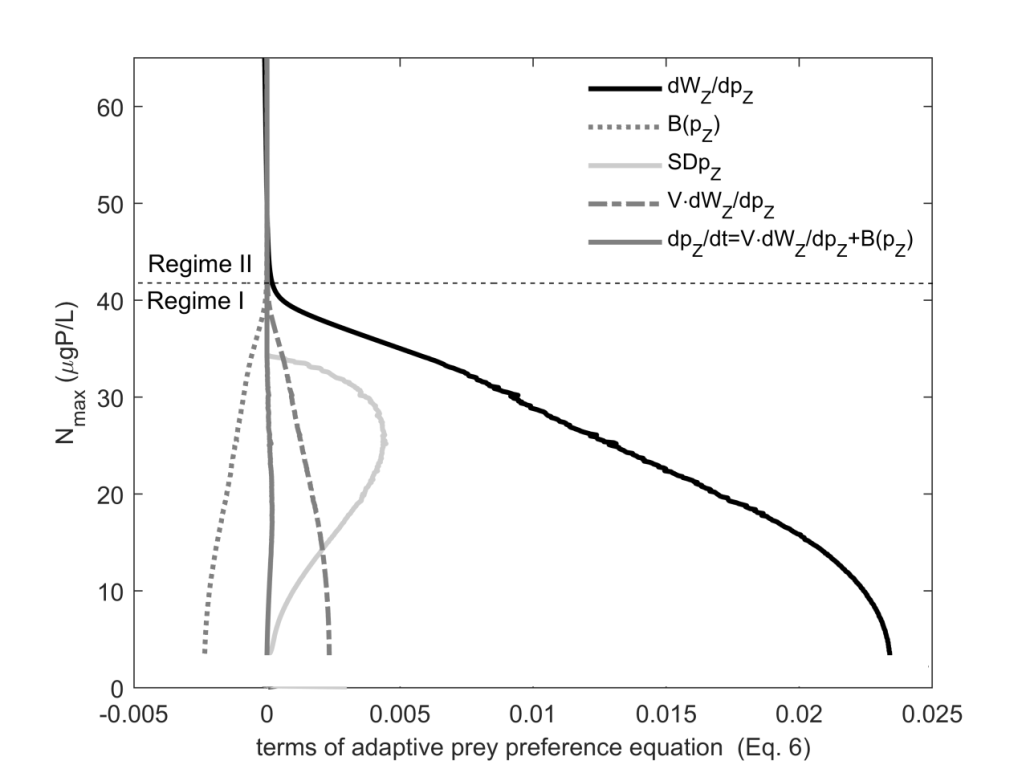


***Figure S3.3*** *Illustration of the values for the different terms of Eq. 6 and the complete equation* $dp_{z}/dt$ *(dark grey solid line), as well as the standard deviation of* $p_{Z}$ *(light grey solid line) along increasing* $N_{max}$*. Illustrated are the fitness term* $dW_{Z}/dp_{z}$ *(black solid line), the boundary function* $B{(p}_{Z})$ *(dotted line), and the complete first term of Eq. 6,* $V\cdot(dW_{Z}/dp_{z})$ *(dashed-dotted line).The grey dashed horizontal line indicates the shift from regime I to II. The values are calculated from mean equilibrium biomass levels.*

**S4. Energy flow pattern dependent on maximum nutrient availability and equilibrium prey preference value**


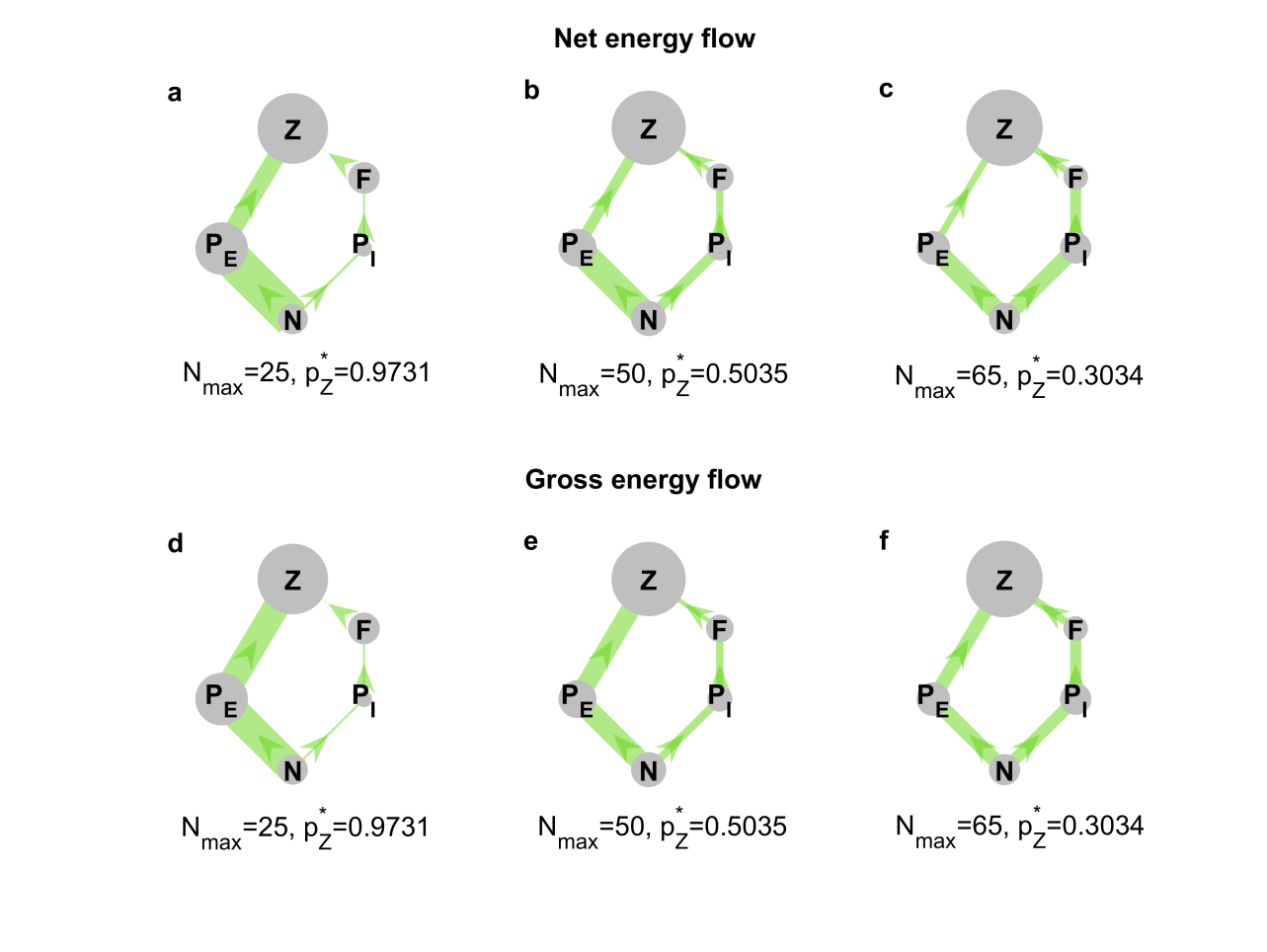


**Figure S4.1** Relative distribution of energy flow for the adaptive preference case at different values of $N_{max}$ and $p_{Z}^{*}$, illustrated along each interaction pathway are (a-c) net energy gain $g_{P_{E}Z}$, equivalent to turnover rate (production/biomass), and (d-f) gross energy flow $\frac{g_{P_{E}Z}}{e_{P}}$. For each case, the width of connections (green) indicates the relative amount of energy flow along the corresponding connection with respect to total energy flow in the food web.


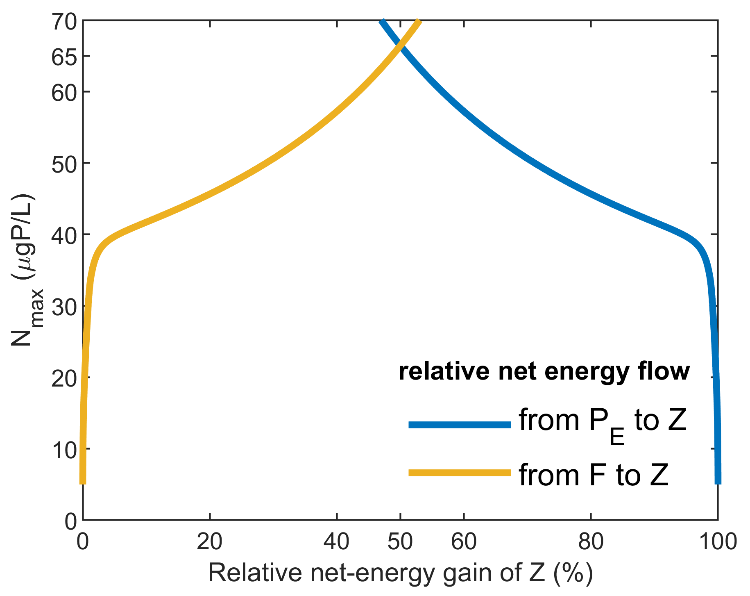


**Figure S4.2** Relative contribution of net energy flow along the links of $P_{E}-Z$ ($g_{P_{E}Z}$, blue) and the $F-Z (g_{FZ},$orange) for the adaptive preference case. Energy flow dominates along the $P_{E}-Z$ link for $N_{max}$ < 66.6, but shifts to dominance along the $F-Z$ link for $N_{max}$ > 66.6.

**S5. The influence of assumptions on the parameterization and functional response type for energy flow and biomass changes with nutrient enrichment**

**Abrupt shift in prey preference creates a bottleneck in energy flow**

As mentioned in the main manuscript, the mycoloop food web is structurally equivalent to the central plankton food web, consisting of two groups of phytoplankton (small and large), a herbivore (ciliate) feeding on small phytoplankton and a selective top predator (copepod) feeding on the ciliate and the large phytoplankton (Thingstad and Sakshaug 1990, Stibor et al. 2004, Wollrab and Diehl 2015). Thereby, the ciliates in the plankton food web are structurally at the same position as the parasitic fungi in the mycoloop food web. Both food webs exhibit a pronounced shift in community composition and response along a nutrient gradient. Due to its structural equivalence, a comparison between dynamic properties of the plankton and the mycoloop food web can provide insight on the interplay of structural features, functional response type and parameterization and its indications for the occurrence of abrupt regime shifts along a nutrient gradient.

Following the food web response with nutrient enrichment for both food webs (Fig. 2 and Wollrab and Diehl 2015), the phytoplankton which is directly consumed by zooplankton is predicted to decrease while ciliates/fungi increase. For both webs this leads to an increasing contribution of ciliates/fungi to the diet of the top consumer with increasing nutrient availability. Under the assumption of a functional response type III at the top consumer level, an abrupt regime shift from dominance of energy flow along the direct phytoplankton-zooplankton pathway to the ciliate/fungi mediated pathway emerging from non-edible phytoplankton at higher nutrient availability can be observed.

**Mechanisms for occurring a drastic shift in community response**

Notably, in both cases this abrupt shift in prey preference creates a bottleneck in energy flow and leads to a corresponding drastic shift in community response to further enrichment. Thereby, the bottleneck arises through different mechanisms: energy transport via the mycoloop is limited by the much lower infection (production) rate of fungi compared to the growth rate of edible phytoplankton. Accordingly, for the mycoloop web the regime shift does not occur if the productivity rate of fungi is equal to edible phytoplankton (Fig. S5.1a). In the plankton food web, the saturation of the ciliate food uptake rate (which follows a type II functional response), due to high prey availability, limits the energy flow to zooplankton (Wollrab and Diehl 2015).

For both webs the occurrence of the regime shift critically depends on the assumption of a functional response type III at the top consumer level and does not occur if a functional response type II is assumed (Fig. S5.2, and Wollrab and Diehl 2015). For the mycoloop food web the regime shift only occurs for the adaptive consumer with type III functional response, but not for the non-adaptive (fixed preference) case. For the plankton food web the occurrence of a regime shift is absent if a linear food uptake term (no saturation) is assumed for the ciliates (Wollrab and Diehl 2015). For the mycoloop web the functional response type assumed for fungal infection does not critically affect the principle occurrence of a regime shift. Under the assumption of a functional response type II for the dependence of fungal infection on host abundance, the regime shift occurs for lower *N_max_* levels compared to the default case and leads to a strong dominance of inedible phytoplankton at higher *N_max_* levels (Fig. S5b).

These results indicate that a selective multispecies predator in combination with either high differences in production rates between alternative prey or non-linear food uptake terms of prey species have a high potential for abrupt regime shifts with nutrient enrichment.


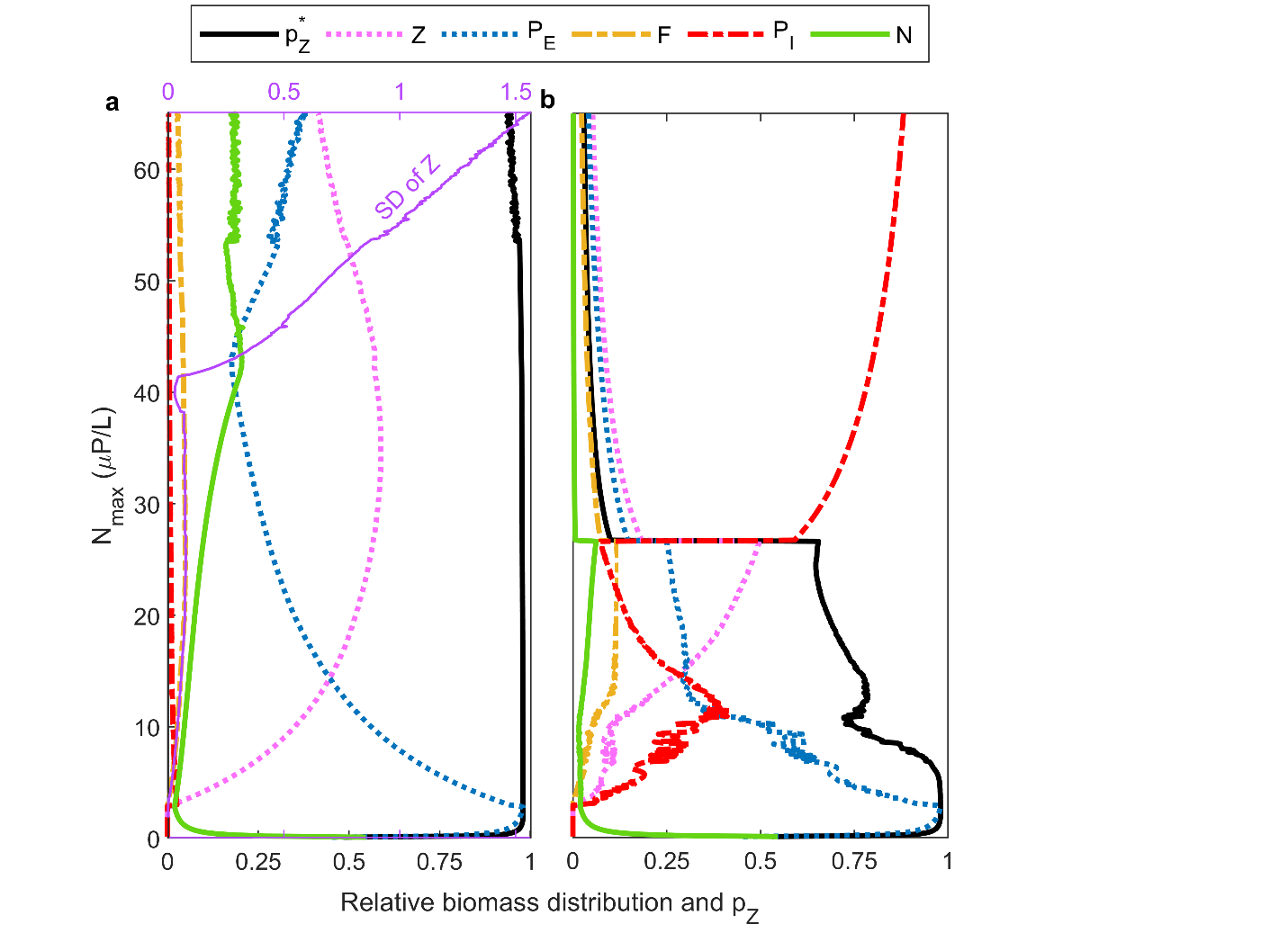


***Figure S5.1*** *Community response pattern along the enrichment gradient for the adaptive preference case, for (a) β =* $\mu_{max,E}$*, and (b) the infection of* $F$ *on* $P_{I}$ *following a non-linear relationship (functional response type II)* with half-saturation constant $K$ (see Table S1) $\left( \frac{\beta P_{I}}{K+P_{I}}F \right)$ *and β identical to the default parameterization (β* $\ll\mu_{max,E}$*).*

**
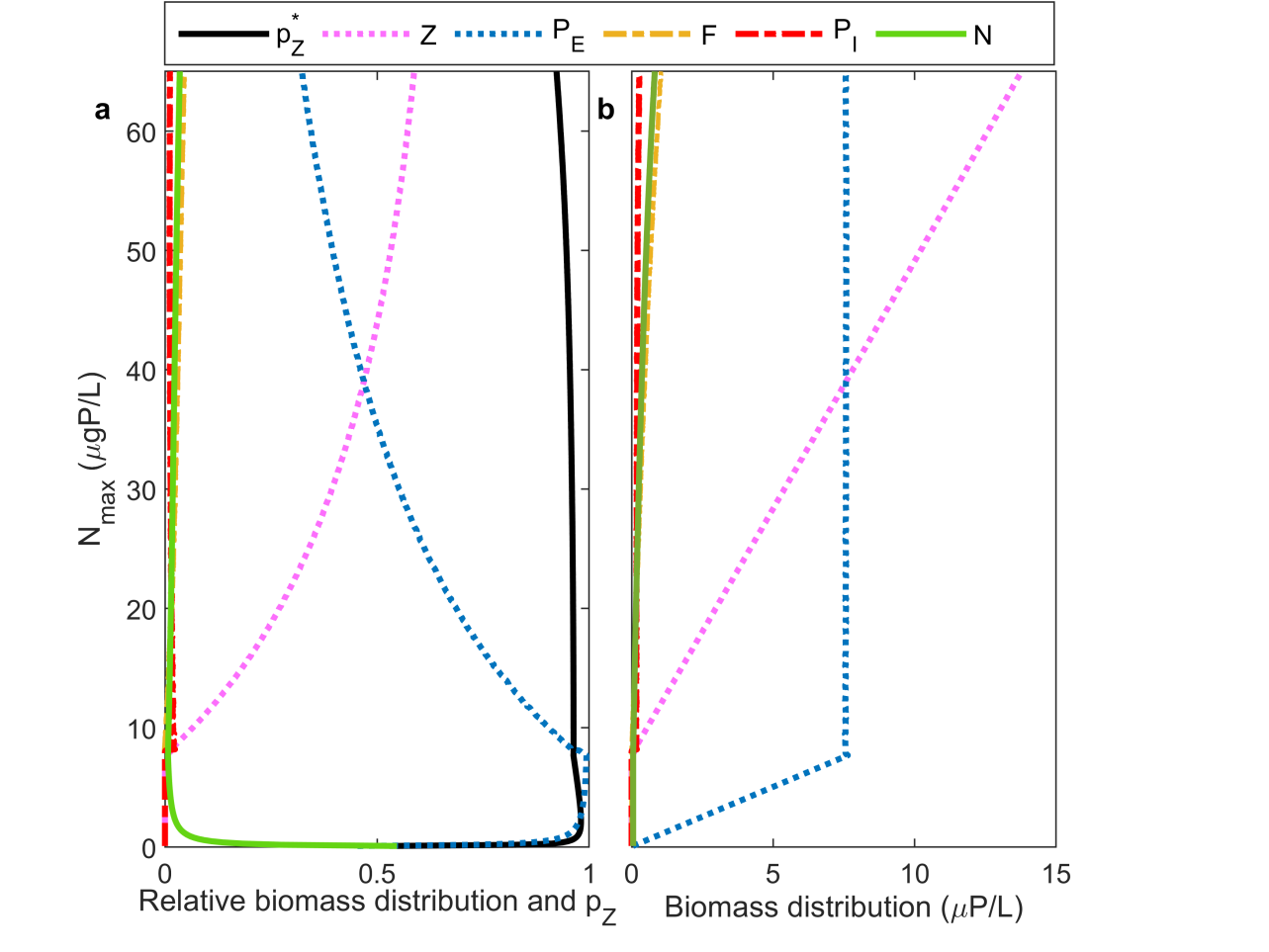
**

***Figure S5.2*** *Community response pattern to nutrient enrichment (increasing* $N_{max}$*) for the adaptive preference case assuming a functional response type II for zooplankton. For this the* the food uptake term of zooplankton in Eqs 2, 4 and 5 was changed to $\frac{\left( e_{p}\cdot p_{Z}\cdot a_{Z}\cdot P_{E}+e_{F}\cdot\left( 1-p_{Z} \right)\cdot a_{Z}\cdot F \right)}{1+h_{P_{E}}\cdot p_{Z}\cdot a_{Z}\cdot P_{E}+h_{F}\cdot\left( 1-p_{Z} \right)\cdot a_{Z}\cdot F}$. *(a) Relative biomass distribution of* $Z$*,* $P_{E}$*,* $F$*,* $P_{I}$ *and* $N$ *as well as optimal preference* $p_{Z}$*, (b) absolute biomass distribution.*
